# Current trends and future projection for addressing the burden of esophageal carcinoma in Asia: a comprehensive analysis (1990–2040)

**DOI:** 10.3389/fonc.2025.1587846

**Published:** 2025-06-23

**Authors:** Peng Ma, Xiaohong Tan, Tingting Hao, Pengfei Liu, Min Bai, Lei Dong

**Affiliations:** ^1^ Gastroenterology, The Second Affiliated Hospital of Xi’an Jiaotong University, Xi’an, China; ^2^ Gastroenterology, Yanan University Affiliated Hospital, Yan’an, China; ^3^ Department of Medicine, Yan’an Vocational and Technical College, Yan’an, China

**Keywords:** esophageal carcinoma, global burden of disease, Asia, current trends, projection

## Abstract

**Background:**

Esophageal carcinoma (EC) significantly impacts global health, particularly in Asia, where many low- and middle-income countries face substantial burdens despite advancements in some regions.

**Objective:**

This study analyzed EC’s spatial and temporal distribution in Asia using the Global Burden of Disease (GBD) database, aiming to forecast future burdens and support effective prevention strategies.

**Methods:**

Data from 48 Asian countries (1990-2021) were extracted from the GBD database, covering incidence, prevalence, deaths, disability-adjusted life years (DALYs), risk factors, and socio-demographic index (SDI). R and GraphPad Prism were used to assess changes and predict future trends.

**Results:**

From 1990 to 2021, EC’s disease burden in Asia generally declined, with significant regional and sex disparities. East Asia showed the most improvement despite having the highest burden. Conversely, South and Southeast Asia experienced limited progress, with some areas seeing increased burdens. Males consistently had higher burdens than females, especially in East Asia. Future projections (from 2022 to 2040) suggested a slight rise in incidence in East Asia, while improvements in South and Southeast Asia may remain limited, though an overall burden decline was expected.

**Conclusion:**

The reduction in Asia’s EC burden underscored the impact of medical advances and public health efforts, but regional and sex disparities persist. Future strategies should enhance health resources in under-resourced and high-risk areas and implement targeted policies to address health inequalities and promote balanced public health development across Asia.

## Introduction

Esophageal carcinoma (EC) is one of the leading malignant tumors worldwide, characterized by high morbidity and mortality, and it poses a significant public health challenge among gastrointestinal cancers (1). Data from the Global Burden of Disease (GBD) database reveal notable variations in the incidence and mortality of EC based on geography, sex, and so on (2). Globally, the disease burden is particularly pronounced in the Asian region (3, 4), especially in East and Central Asia, where the majority of global cases are reported. The high prevalence of EC in Asia is likely associated with a combination of factors, including dietary habits, lifestyle choices, genetic predisposition, and environmental exposures (5). Contributing factors such as long-term smoking, excessive alcohol consumption, poor dietary practices, and potential infectious agents like Helicobacter pylori (6) or human papillomavirus (HPV) also play a significant role in the elevated incidence rates observed in the region.

The GBD database serves as a crucial resource for studying the spatial-temporal distribution trends of disease burden. It enables systematic analysis of key indicators such as the incidence, mortality, and disability-adjusted life years (DALYs) associated with EC, as well as the regional disparities in disease burden (7). Additionally, long-term trend analysis and predictive modeling based on GBD data can provide valuable insights into future changes in disease burden, offering a scientific foundation for the development of effective public health policies. However, there is a notable lack of systematic studies utilizing the GBD database to examine the disease burden of EC in Asia, particularly in terms of dynamic trends and future projections. This gap underscores the need for further research in this area to better inform regional health strategies.

Thus, this study utilized the GBD database to systematically analyze the spatial-temporal distribution characteristics of the disease burden associated with EC in Asia. It specifically focused on historical trends in incidence, mortality, and DALYs. Additionally, the study projected the future disease burden of EC to provide scientific evidence and data-driven support for the formulation of prevention and control strategies in Asia.

## Methods

### Data collection

Drawing from the GBD 2021 (https://vizhub.healthdata.org/gbd-results/) database, the current study collected epidemiological data on EC in Asia, including incidence, prevalence, mortality, DALYs, EC-related risk factors (Diet low in vegetables: the daily vegetable intake fell short of the recommended minimum level (200–300 g per day); Chewing tobacco: using chewed tobacco products, including but not limited to tobacco leaves, tobacco pellets, and other oral tobacco products; High alcohol use: the daily consumption of alcohol exceeds the safe limits recommended by national and international health organizations (daily pure alcohol intake ≥ 60 g in male and ≥ 40 g in female); Smoking: the act of using tobacco products, e.g. cigarettes, cigars, pipes, etc.) and Socio-Demographic Index (SDI) of 48 countries (**Supplementary Table 1**) belonged to Asia. By analyzing data spanning the period from 1990 to 2021, the study offered a comprehensive evaluation of the disease burden of EC across Asia and its individual countries, while also examining its temporal trends over time.

### Data analysis

#### Parameters

##### Fixed parameters

###### Risk factor

We assumed current age-specific exposure levels would remain stable, as no future behavioral trends were incorporated.

##### Dynamic parameters

Demographic Shifts: Population structure projections were derived from IHME 2017–2100 data, accounting for fertility, mortality, and migration trends.

#### Statistical effects

##### Age effect

Age-specific EC risks were adjusted for anticipated shifts.

##### Period effect

The overall level of risk in a given year.

##### Cohort effect

Birth cohort-specific risks were included using APC (Age-Period-Cohort) models.

#### Tools

R (version 4.4.1) and GraphPad Prism (version 10.1.2) were used for data analysis and visualization. In brief, the “ggplot2” package was utilized to visualize the trends in the disease burden across Asia. To map the degree of change and the Estimated Annual Percentage Change (EAPC) in Asia, the “maps” and “ggplot2” R packages were employed. Additionally, GraphPad Prism 10 software was used to illustrate the percentage contribution of various risk factors over different years in China. The relationship between the SDI and age-standardized death rates (ASDR) for EC was visualized using the “ggprepel” package. Furthermore, the “BAPC” and “ggplot2” R packages were applied to project and chart forecasted data for each region in Asia from 2022 to 2040. In brief, we employed the Bayesian age-period-cohort (BAPC) model, which is grounded in the core assumption that historical trends in disease rates would persist in a similar pattern. The model incorporated two types of dynamic changes: demographic change (based on the IHME population projection data from 2017 to 2100) and three statistical effects: age effect (reflecting risk differences between age groups), period effect (capturing the overall risk of the year), and cohort effect (representing the characteristics of the birth cohort). Predictive power was evaluated through retrospective validation, and uncertainty was quantified using 95% confidence intervals. To ensure applicability to region-specific disease patterns, the models were independently developed for the four Asian regions.

## Results

### The trends of disease burden of EC in Asia

**Figure 1:**
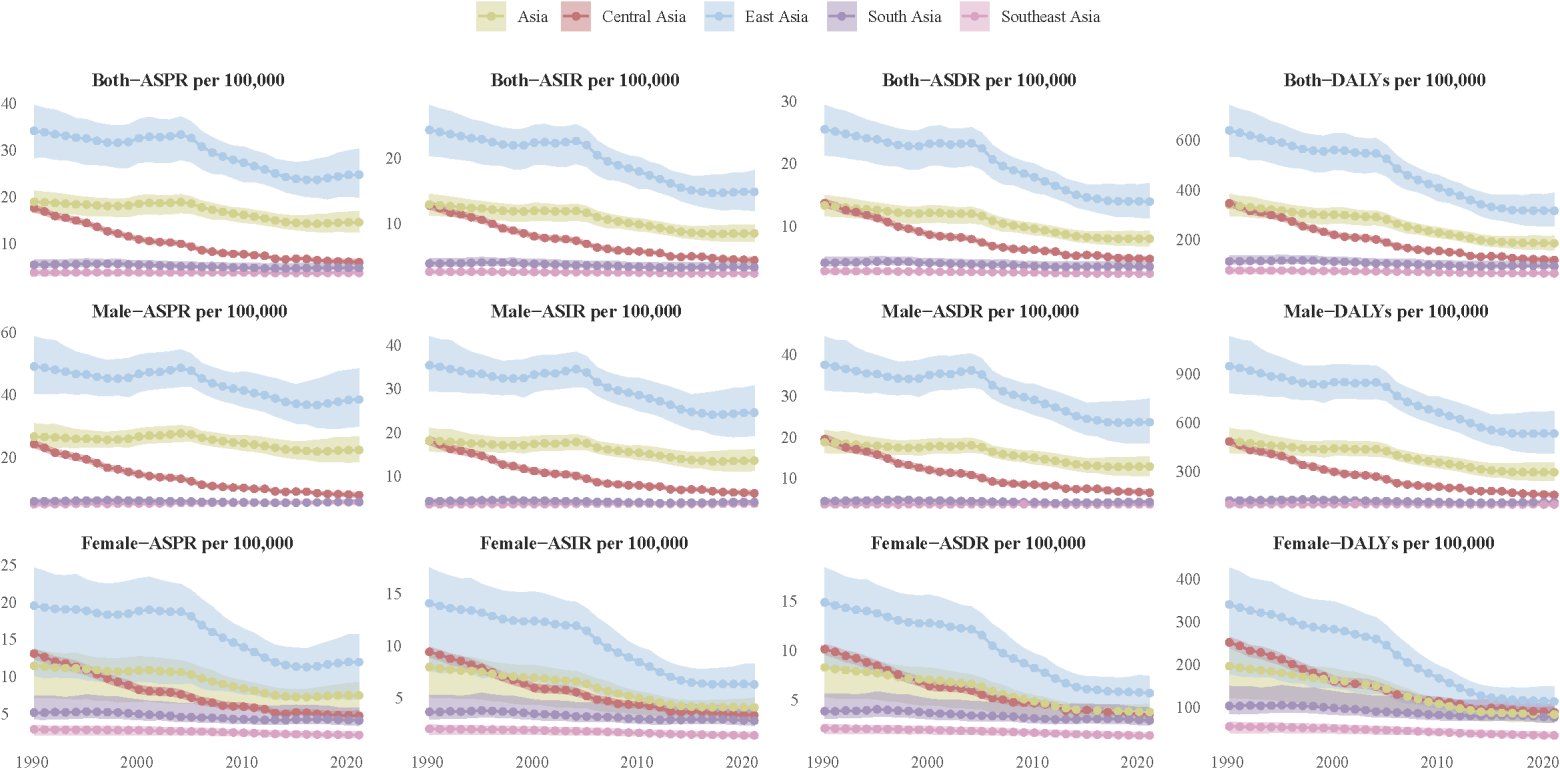
The disease burden of esophageal carcinoma (EC) in Asia from 1990 to 2021. The figure was divided into six panels illustrating both sexes (top row), males (middle row), and females (bottom row) for ASPR, ASIR and DALYs. ASPR on the left, ASIR in the middle, and DALYs on the right. Each panel displayed the rates per 100,000 population, with distinct color coding for regions. Shaded areas represent the confidence intervals for the estimates. The trends highlight variations in health outcomes across regions and genders over the three decades, providing insight into the changing health landscape in Asia. Abbreviation: ASPR, Age-Standardized Prevalence Rate; ASIR, Age-Standardized Incidence Rate; ASDR, Age-Standardized Deaths Rate; DALYs, Disability-Adjusted Life Years.

The analysis of the data revealed that the disease burden of EC in Asia, including age-standardized prevalence rate (ASPR), age-standardized incidence rate (ASIR), ASDR, and DALYs, exhibited a significant overall downward trend from 1990 to 2021. However, notable regional and sex differences were observed. While East Asia consistently bore the highest disease burden, it also showed the most substantial decline. In contrast, South and Central Asia experienced lower overall disease burdens, but the decline was relatively modest. Southeast Asia had the lowest and most stable burden of EC throughout the period. From a sex perspective, males exhibited significantly higher ASPR, ASIR, and ASDR compared to females, with East Asian males being particularly affected. Although the disease burden in females was generally lower, regional disparities remained evident (**Figure 1**, **Supplementary Table 2**).

### The changes of disease burden of EC in Asia

**Figure 2:**
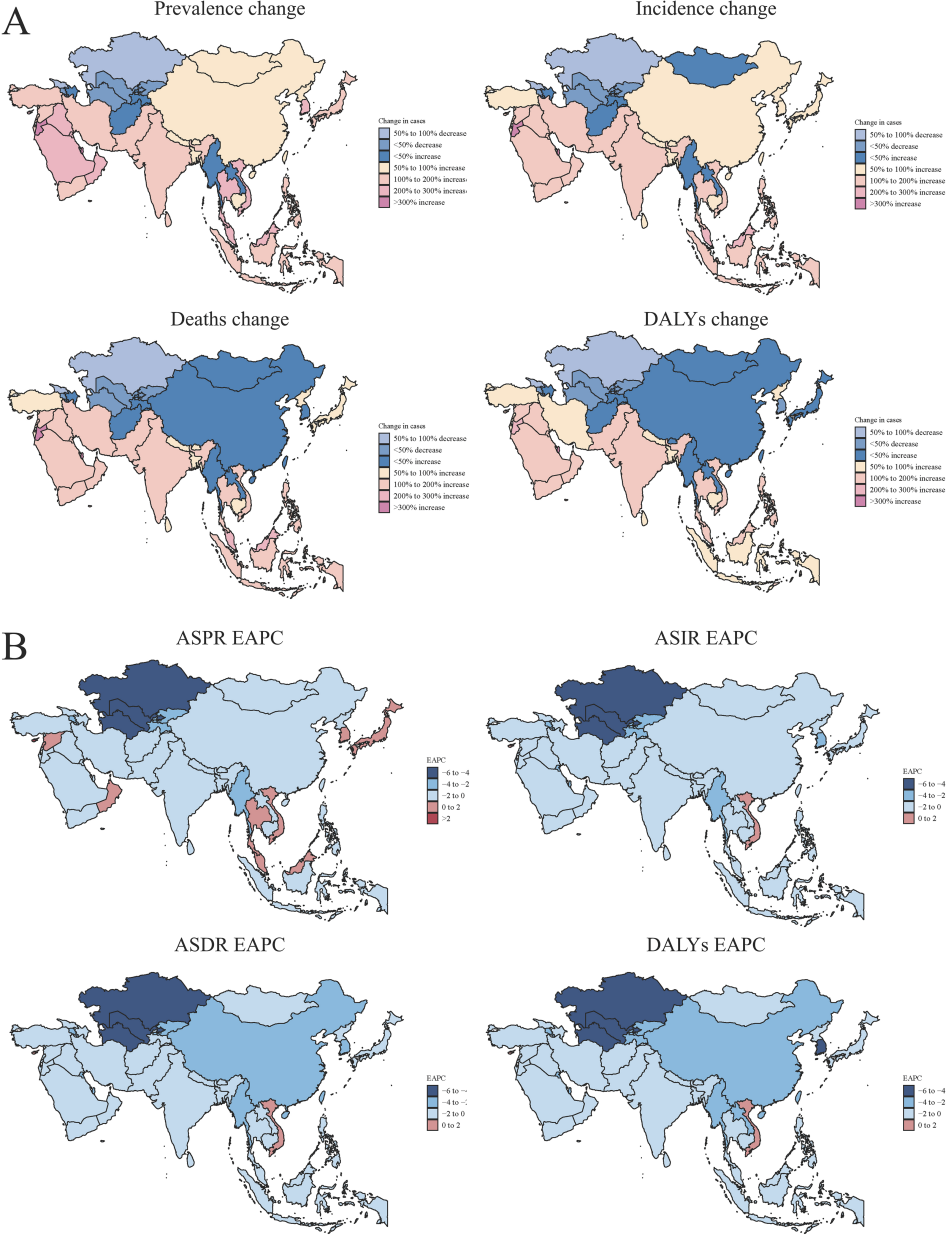
The changes of disease burden of EC in Asia. **(A)** The percentages of changes in the number of prevalence, incidence, deaths and DALYs. Prevalence change (top left): Indicates the variation in disease prevalence across different regions. Incidence change (top right): Shows changes in disease incidence. Deaths change (bottom left): Displays changes in deaths rates, differentiated by percentage decrease and increase. DALYs change (bottom right): Represents changes in DALYs, highlighting regions with significant variations. **(B)** The changes in the Estimated Annual Percentage Change (EAPC) of ASPR, ASIR, ASDR and DALYs. ASPR EAPC (top left): indicating regional trends. ASIR EAPC (top right): illustrating the annual percent change across regions. ASDR EAPC (bottom left): highlighting deaths trends. DALYs EAPC (bottom right): Trends in DALYs across Asia.

Between 1990 and 2021, the percentage change in the disease burden of EC in Asia demonstrated significant regional variability. East and Central Asia, including countries such as China, Mongolia, and Kazakhstan, experienced substantial reductions in prevalence, incidence, deaths, and DALYs, with decreases exceeding 50%. In contrast, parts of South and Southeast Asia, such as India, Pakistan, Indonesia, and the Philippines, witnessed an increasing burden of EC. Notably, these regions experienced sharp rises in incidence and DALYs, ranging from 50% to over 200% (**Figure 2A**).

The EAPC of EC in Asia exhibited an overall downward trend. This decline was particularly significant in East and Central Asia, including countries such as China, Mongolia, and Kazakhstan, where substantial reductions were observed in prevalence, incidence, deaths, and DALYs. Conversely, some countries in South and Southeast Asia, such as Thailand, Cambodia, and Myanmar, experienced a positive increase in EAPC, indicating a rising burden of EC (**Figure 2B**).

**Figure 3:**
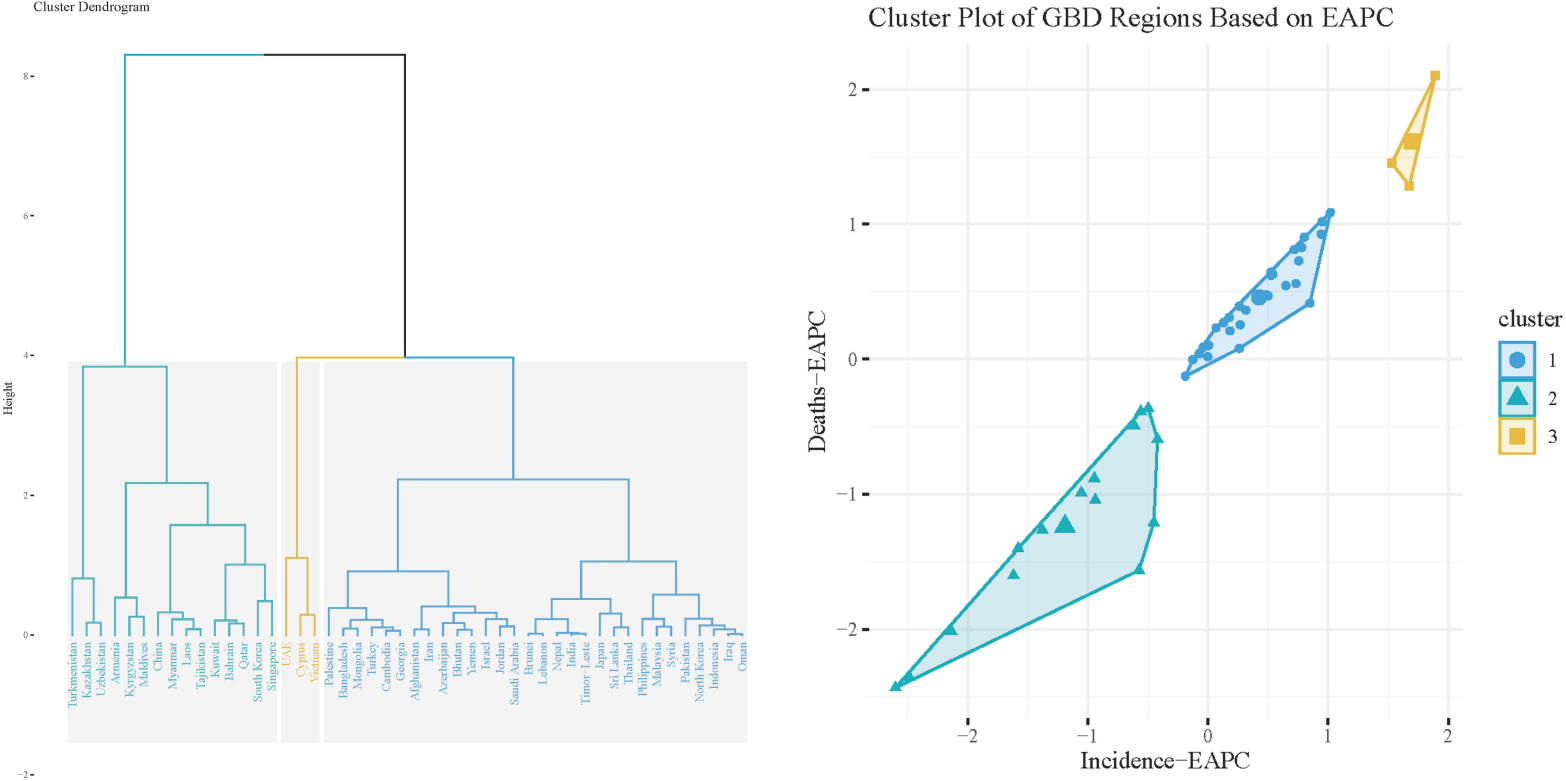
The cluster analysis of EAPC in different Asian countries. Left Panel: Cluster Dendrogram depicting the hierarchical clustering of different countries/regions. The height of the branches represents the distance or dissimilarity between clusters, with closely related regions grouped together. The dendrogram shows the formation of distinct clusters, which can aid in identifying regions with similar health trends. Right Panel: Cluster Plot illustrating the relationship between deaths (Deaths-EAPC) and incidence (Incidence-EAPC) across the identified clusters. Each point represents a country/region, color-coded by cluster.

The cluster analysis of the EAPC in EC across Asia revealed significant differences in the dynamic characteristics of the disease among countries. In Central Asia and certain Southeast Asian nations, such as Turkmenistan and Kazakhstan, the EAPC values demonstrated notably negative growth, suggesting a declining disease burden of EC. Meanwhile, in the Middle East and some Southeast Asian countries, including Singapore and the United Arab Emirates, EAPC values were close to zero, indicating that the disease burden has stabilized. However, in South Asia and certain Southeast Asian nations, such as India and Myanmar, EAPC values showed positive growth, reflecting a continued rise in the disease burden (**Figure 3**).

### The disease burden of EC in different East Asian countries

**Figure 4:**
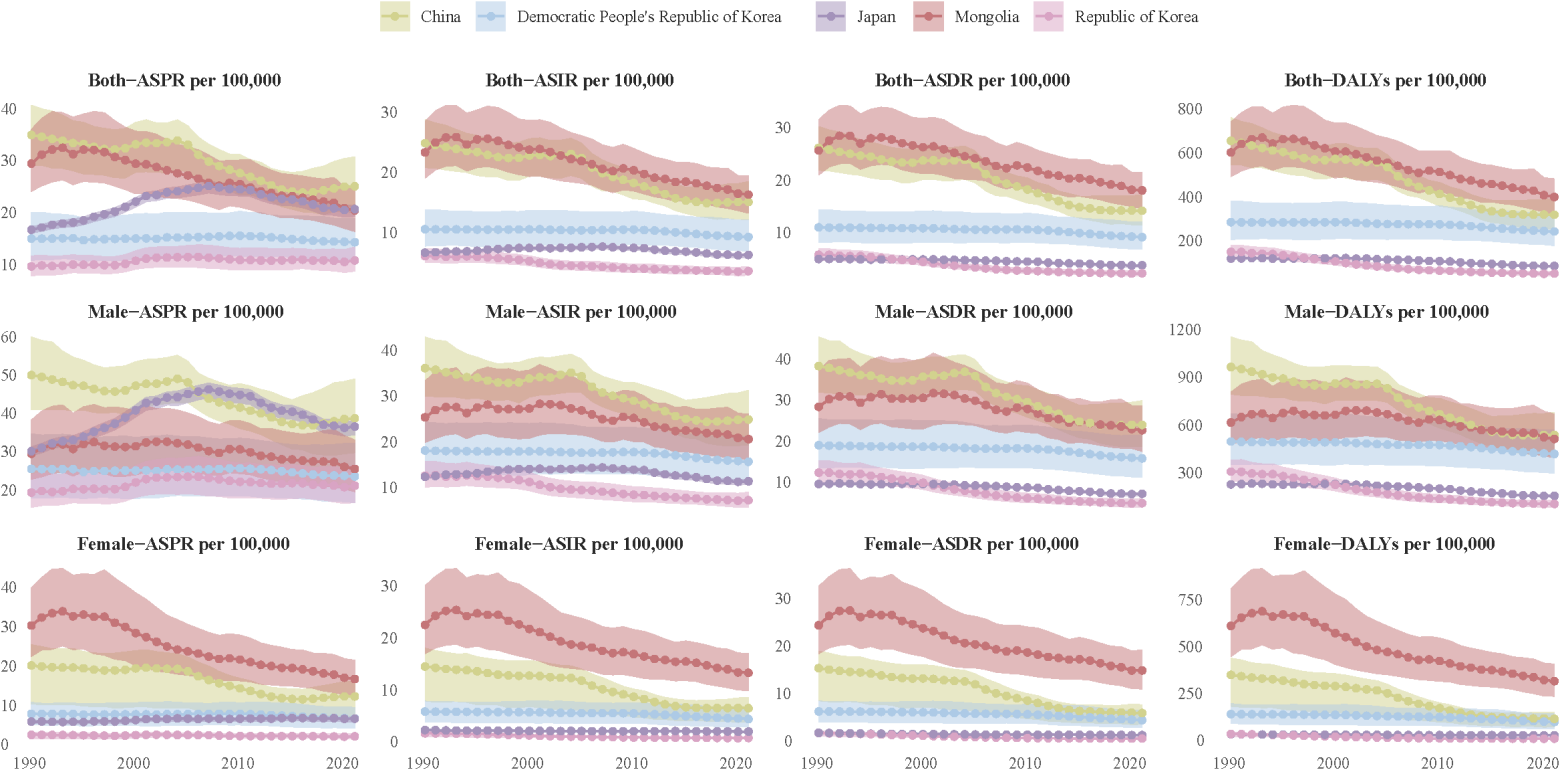
The disease burden of EC in East Asian countries. The figure is divided into 12 panels, each representing a different health metric: ASPR for both sexes (row 1 top), males (row 1 middle), and females (row 1 bottom). ASIR for both sexes (row 2 top), males (row 2 middle), and females (row 2 bottom). ASDR for both sexes (row 3 top), males (row 3 middle), and females (row 3 bottom). DALYs for both sexes (row 4 top), males (row 4 middle), and females (row 4 bottom). Each line represents data from different countries, with color coding. The trends illustrate changes in health outcomes over the specified years, highlighting variations among different countries and between genders.

Previous studies found that the disease burden of EC in East Asia turned a turning point around 2004. To clarify the reasons for this change, this study further analyzed the trends of disease burden of EC in East Asian countries, respectively. From 1990 to 2021, the disease burden of EC in East Asian countries exhibited notable differences and evolving trends. In China, while the ASPR of EC remained high, significant declines were observed in the ASIR, ASDR, and DALYs, with a clear turning point around 2004. In contrast, Mongolia experienced a higher disease burden with only a modest decline over the same period. Meanwhile, Japan and Republic of Korea consistently maintained the lowest burden of EC, with relatively stable trends across all indicators. The Democratic People’s Republic of Korea exhibited a moderate burden of disease, but with slower improvements compared to other countries in the region. Sex disparities in the burden of EC were evident, with males generally experiencing a significantly higher burden than females. This disparity was particularly pronounced in China and Mongolia. In contrast, females had a comparatively lower disease burden, and the downward trend in their indicators was more pronounced and consistent over time (**Figure 4**).

**Figure 5:**
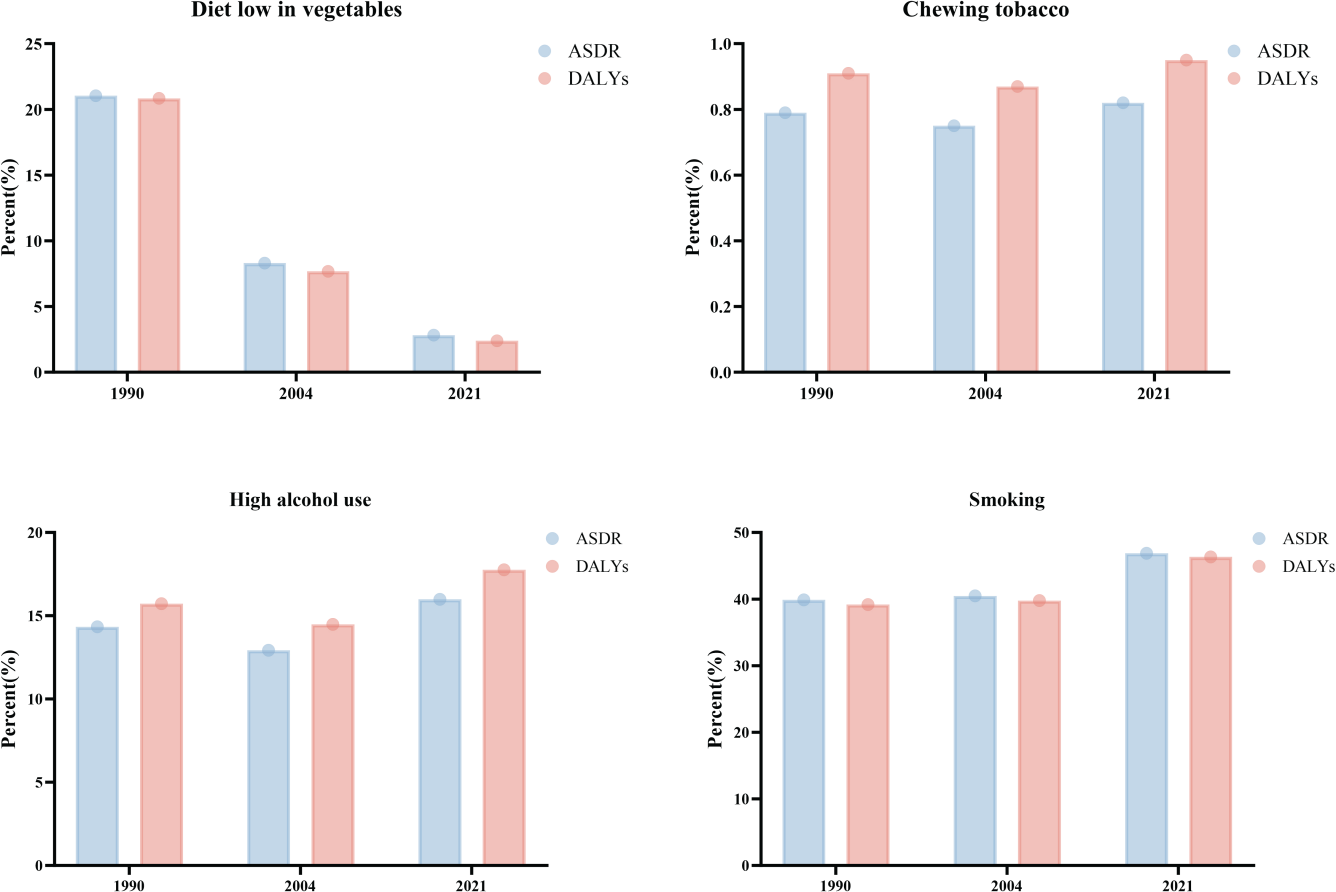
The risk factors of EC in China. The figure comprises four panels, each representing a different health risk factor: Top Left: Diet Low in Vegetables, the blue bars represent the ASDR, while the red bars indicate DALYs. Top Right: Chewing Tobacco. Bottom Left: High Alcohol Use. Bottom Right: Smoking. Each panel provides a visual comparison of health outcomes associated with these risk factors over time, illustrating changes in public health trends in China.

In conclusion, the significant decline in the burden of EC in China around 2004 may be the primary driver behind the changing trends of EC burden in East Asia. To further explore this decline, we analyzed the contribution of various risk factors to EC-related deaths and DALYs in China. The findings revealed that the proportion of deaths and DALYs attributable to a diet low in vegetables decreased dramatically, from over 20% in 1990 to less than 5% in 2021, suggesting that improved dietary habits have substantially reduced the associated risk. However, other risk factors displayed different trends. The proportion attributable to chewing tobacco remained relatively stable over the years, accounting for approximately 0.8% of EC deaths and DALYs. In contrast, the proportion related to high alcohol consumption increased steadily, rising from around 15% in 1990 to nearly 20% in 2021. Smoking, identified as the most significant risk factor, consistently contributed to over 40% of EC deaths and DALYs from 1990 to 2021, with only limited improvement observed (**Figure 5**).

### The relationship between the disease burden of EC and SDI in different countries

**Figure 6:**
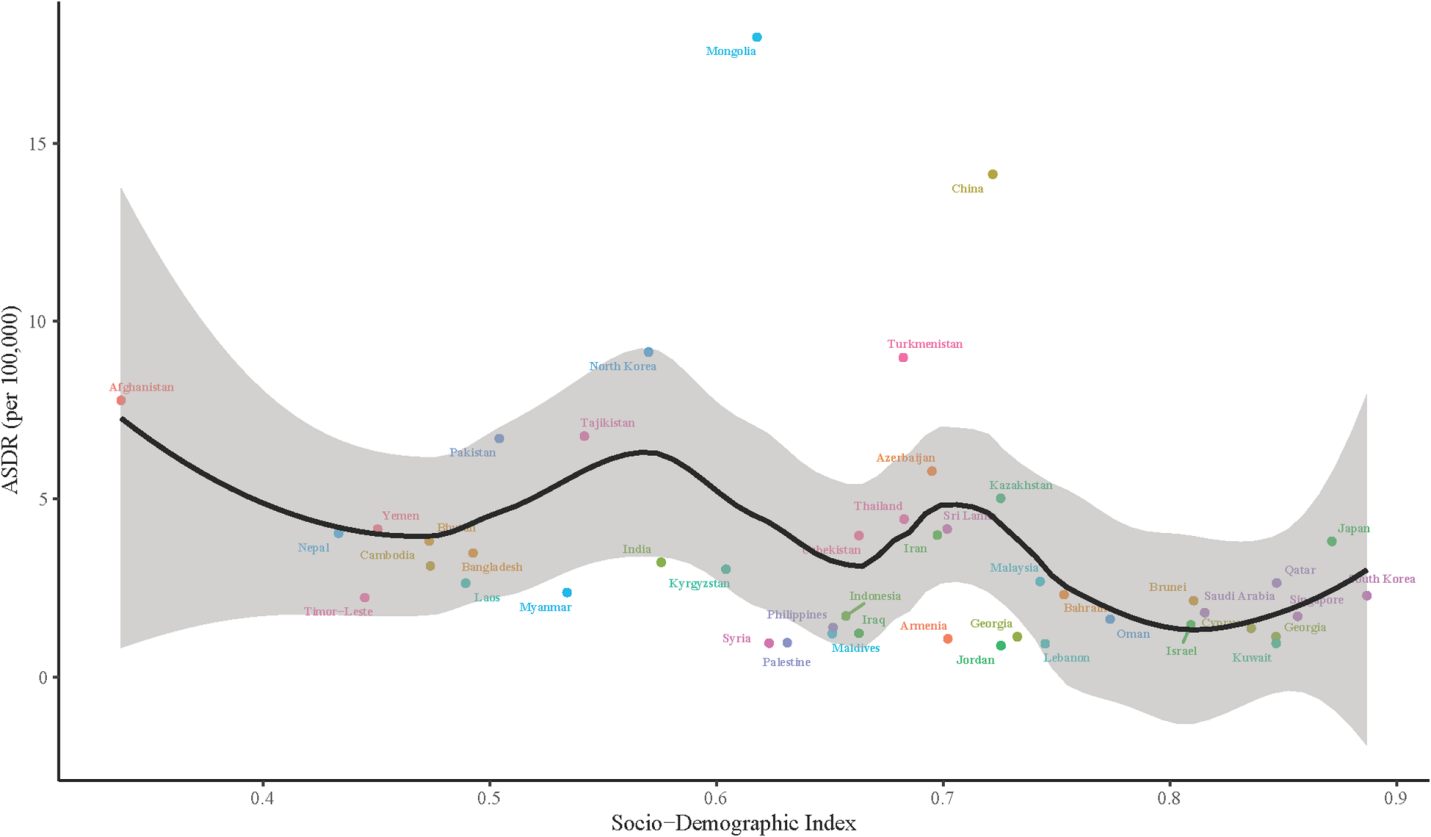
The relationship between ASDR and Socio-Demographic Index (SDI) in Asian countries. The x-axis represents the SDI, ranging from 0 to 1, while the y-axis shows the ASDR per 100,000 population. Each point corresponds to a country, color-coded for identification. A black line indicates the overall trend in ASDR as SDI increases, with a shaded area around the line representing the confidence interval.

There was a non-linear relationship between the ASDR of EC and the SDI in Asian countries, following a U-shaped trend. In countries with low SDI, such as Afghanistan and Nepal, EC ASDR was relatively high, ranging from 5 to 10 per 100,000. As SDI increased to moderate levels, seen in countries like India and Thailand, mortality rates drop significantly, with most falling below 5 deaths per 100,000. However, some countries with moderate SDI, such as China and Turkmenistan, continued to exhibit higher ASDR levels. In high SDI countries, including Japan, South Korea, and Singapore, EC ASDR was generally low, typically under 5 deaths per 100,000. Yet, a slight rebound in ASDR had been observed in a few high SDI nations. Notably, Mongolia stood out with a significantly higher mortality rate—exceeding 15 deaths per 100,000. Overall, ASDR of EC was highest in low SDI countries, lowest in medium SDI countries, and showed a modest upward trend in certain high SDI countries (**Figure 6**).

### The prediction of disease burden of EC in Asia from 2022 to 2040

**Figure 7:**
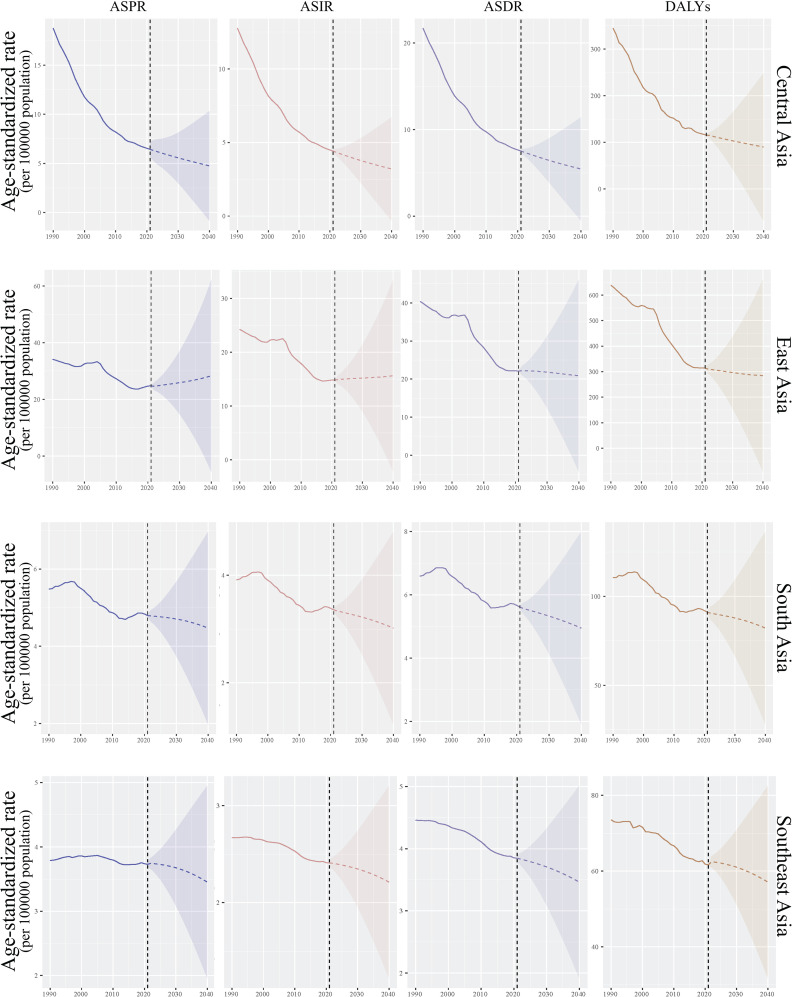
The prediction of disease burden of EC in Asia. The figure consists of four columns, each representing a specific health metric (from left to right): ASPR, ASIR, ASDR and DALYs. The rows are divided into four geographical regions (from top to bottom): Central Asia, East Asia, South Asia and Southeast Asia. Each panel includes a shaded area representing the confidence interval around the trend line. This figure illustrates the trends in health metrics over time, highlighting variations in health outcomes across different regions in Asia.

From 2022 to 2040, the disease burden of EC across the four regions of Asia was projected to follow an overall downward trend, although the extent of improvement will vary significantly between regions. Central Asia was expected to achieve the most substantial progress, with all key indicators showing marked improvement. The ASPR was forecasted to decline from 6.4 (95% confidence interval (CI): 5.7-7.2) per 100,000 to 4.7 (95%CI: -0.8-10.3) per 100,000, reflecting notable success in the prevention and control of EC in this region. East Asia presented a more complex scenario. While ASDR and DALYs were expected to decrease (from 22.1 (95%CI: 22.0-22.2) to 20.9 (95%CI: -4.4-46.1) per 100,000 for ASDR and from 313.9 (95%CI: 313.7-314.2) to 284.7 (95%CI: -92.2-661.6) per 100,000 for DALYs), ASPR and ASIR were projected to rise slightly, from 24.7 (95%CI: 24.6-24.8) to 28.3 (95%CI: -5.6-62.3) and from 14.8 (95%CI: 14.8-14.9) to 15.6 (95%CI: -2.3-33.5) per 100,000, respectively. These trends suggested an improvement in treatment outcomes and patient survival rates in the region. In South Asia and Southeast Asia, reductions in the EC burden were expected to be more modest. ASPR was projected to decline slightly, from 4.8 (95%CI: 4.7-4.8) to 4.5 (95%CI: 2.0-7.0) per 100,000 in South Asia and from 3.7 (95%CI: 3.7-3.8) to 3.5 (95%CI: 2.0-5.0) per 100,000 in Southeast Asia. In summary, while the disease burden of EC was expected to decrease across Asia, the degree of improvement will differ significantly. Central Asia was poised to make the greatest strides, but challenges in East Asia, South Asia, and Southeast Asia—particularly in addressing rising incidence and resource constraints—underscored the need for targeted efforts to reduce disparities and improve outcomes (**Figure 7**).

## Discussion

In current study, we found that, from 1990 to 2021, the disease burden of EC (including ASPR, ASIR, ASDR, and DALYs) in Asia showed an overall downward trend, though notable regional and sex disparities persisted. The burden was highest—but saw the most significant improvement—in East Asia, while progress was limited in Central and South Asia. In contrast, Southeast Asia maintained the lowest and relatively stable burden. Males experienced a significantly higher disease burden than females, particularly in East Asia, where the disparity was most pronounced. In China, the burden decreased notably around 2004, largely due to dietary improvements; however, smoking and high alcohol consumption remain the primary risk factors. Countries with low SDI faced the highest burden, while medium SDI countries experienced a significant decline. High SDI countries reported the lowest burden overall, though some areas saw a slight rebound. Future projections indicated that the overall burden of EC will continue to decline, with Central Asia expected to see the most dramatic improvements. However, ASIR was likely to rise in East Asia, and progress may remain limited in South and Southeast Asia. Strengthening interventions aimed at reducing risk factors such as smoking and alcohol, along with enhancing healthcare resources and expanding screening coverage, will be crucial for further reducing the disease burden.

### Regional disparities

The overall disease burden of EC in Asia showed a downward trend from 1990 to 2021. However, notable regional and sex disparities persisted. East Asia, while carrying the highest burden (8), achieved significant progress through preventive measures and large-scale screening programs. In contrast, Central and South Asia experienced slower declines, largely due to limited healthcare resources, while Southeast Asia maintained the lowest and relatively stable burden. Additionally, the ASPR and ASDR of EC remained significantly higher in males than in females (9). This disparity was particularly evident among males in East Asia, where high-risk behaviors such as smoking and alcohol consumption were major contributing factors. Looking ahead, efforts must focus on expanding access to healthcare resources, promoting healthier lifestyles, and implementing targeted interventions for high-risk populations (10). Drawing lessons from successful initiatives, such as China’s screening programs and South Korea’s tobacco control policies, can help address regional and sex imbalances. By doing so, the burden of EC can be further reduced, fostering greater health equity across Asia.

### Divergent trends

Over the past 30 years, the changes in the burden of EC across different regions have varied significantly. In East and Central Asia (e.g., China, Mongolia, Kazakhstan), there have been notable reductions of over 50% in all key measures of disease burden. These improvements reflect the success of enhanced medical services and public health interventions in these areas. Conversely, several countries in South and Southeast Asia, such as India, Indonesia, Pakistan, and the Philippines, were encountering a growing burden of EC (11). Increases in ASIR and DALYs in these countries have reached alarming levels, ranging from 50% to 200%. This rising trend was likely linked to insufficient medical resources, low screening coverage, and greater exposure to risk factors. Additionally, analysis of the EAPC revealed positive EAPC values in some Southeast Asian countries, including Thailand, Cambodia, and Myanmar, indicating a continued rise in the disease burden in these regions. These disparities underscored the urgent need to enhance healthcare infrastructure and implement stronger public health interventions, particularly in regions where the burden of EC was on the rise (12).

### Risk factors

From 1990 to 2021, the burden of EC in East Asian countries underwent significant and dynamic changes. In China, the disease burden decreased markedly, particularly after 2004, with notable improvements in ASIR, ASDR, and DALYs. This positive trend could be attributed to shifts in dietary habits, such as increased vegetable consumption, as well as advancements in medical care (13). However, smoking and high alcohol consumption remain major risk factors (14, 15), contributing to approximately 40% of deaths and 20% of DALYs, underscoring the need for targeted interventions. In contrast, Mongolia continued to experience a high burden of EC with limited progress, likely influenced by unhealthy dietary practices or environmental factors. Meanwhile, Japan and South Korea reported the lowest and most stable burden levels in the region, largely due to their advanced healthcare systems and effective risk control measures. On the other hand, North Korea exhibited a moderate disease burden, but the rate of improvement has been relatively slow. A particularly striking trend in East Asia was the pronounced sex disparity, with males generally experiencing a significantly higher burden than females. However, the degree of improvement over time had been greater among females. Given these dynamic changes across East Asia, it was essential to strengthen health intervention policies targeting high-risk populations and to optimize the allocation of medical resources to further reduce the burden of EC in the region (16).

### Socio-demographic influences

The observed U-shaped relationship between EC burden and SDI underscores the critical need for region-specific intervention strategies. In low-SDI countries, where limited medical resources and insufficient health awareness contribute to persistently high ASDR (17), targeted measures should extend beyond general infrastructure improvements (18, 19). Primary prevention efforts could include implementing tobacco and alcohol control policies such as taxation and advertising bans, particularly in high-consumption regions like parts of Africa and South Asia. Public health campaigns focused on known risk factors like hot beverage consumption and betel quid chewing, delivered through community engagement, could further reduce incidence. Secondary prevention strategies should prioritize accessible early detection, including low-cost screening programs using balloon cytology or portable endoscopy in high-risk areas, coupled with training primary healthcare workers to improve symptom recognition and referral efficiency. Moderate-SDI countries, despite showing overall progress, continue to face challenges from persistent risk behaviors. In regions like Central Asia and North Africa, unhealthy dietary habits involving high salt-preserved foods necessitate nutritional policy reforms such as food labeling initiatives and subsidies for fresh produce. Strengthening enforcement of existing tobacco and alcohol regulations could address another major contributor to EC burden in these settings. High-SDI countries with rebounding disease burdens (20, 21), particularly in Western Europe and Japan, require interventions tailored to aging populations and lifestyle factors (22, 23). Healthcare systems should adapt through geriatric oncology programs to optimize treatment for older patients and telemedicine follow-ups to enhance post-treatment surveillance. Behavioral counseling targeting high-risk groups like long-term smokers and heavy drinkers, combined with policy incentives for healthier diets through measures like sugar/alcohol taxes and urban “healthy food zones,” could help mitigate lifestyle-related risks. These findings highlight the importance of moving beyond broad recommendations toward precision public health approaches that account for regional variations in dominant risk factors. While infrastructure and education remain fundamental, successful EC burden reduction will depend on locally adapted interventions addressing specific challenges - whether tobacco use in low-SDI regions or aging populations in high-SDI countries. Future research should evaluate the cost-effectiveness of these proposed strategies across diverse settings to inform policy decisions and resource allocation.

### Future interventions

Over the next 20 years, the disease burden of EC in Asia was expected to continue declining, though the extent of improvement will vary significantly across regions. Central Asia was projected to experience the greatest progress, reflecting the effectiveness of implemented prevention and control measures. In East Asia, the ASDR and DALYs were anticipated to decrease. However, the ASPR and ASIR were expected to rise slightly. This may be attributed to advancements in treatment that improve survival rates, although the increasing number of new cases highlights the need for continued vigilance. In contrast, South and Southeast Asia were projected to see less pronounced improvements. In these regions, ASPR was expected to decline only marginally. This limited progress was closely linked to insufficient investment in medical resources and inadequate health intervention measures. To achieve a comprehensive reduction in the disease burden of EC, it was critical to develop region-specific prevention and control strategies. High-burden areas required targeted investments in medical resources and enhanced public health initiatives, while regions with improving survival rates should focus on addressing the rising number of new cases. A tailored approach will be essential to ensure sustained progress across the diverse healthcare landscapes of Asia.

### Limitations

This study has several limitations that should be considered when interpreting the findings. For example, the ecological nature of GBD data may introduce biases, as it aggregates population-level estimates rather than individual-level data. This could obscure subnational variations in EC burden. Data quality and completeness vary across countries, particularly in regions with underdeveloped health information systems (e.g., parts of Africa and South Asia). Missing or delayed reporting may affect the accuracy of estimates (24). Moreover, future burden projections rely on historical trends and assume continuity in risk factors and healthcare policies. However, unforeseen changes, such as new screening programs, shifts in tobacco/alcohol consumption, or advancements in treatment, could alter trajectories. The model’s dependence on the Socio-demographic Index (SDI) may oversimplify complex interactions between development and disease burden. While key risk factors were included, the study did not comprehensively assess interactions between environmental, genetic predispositions, and dietary habits (25). Qualitative drivers were not examined, which could explain regional disparities beyond quantitative metrics. These limitations suggest that while our findings highlight broad trends, targeted studies—incorporating primary data collection, subnational analyses, and mixed-methods approaches—are needed to refine actionable policies, especially in high-burden regions.

The findings indicated that the decline in the disease burden of EC in Asia was closely linked to levels of social development, the equitable allocation of medical resources, and the implementation of effective health interventions. However, the significant regional and sex disparities highlighted the need for tailored policies and targeted measures to address these variations. In high-burden regions, the focus should be on intensifying early screening and prevention efforts to mitigate the projected rise in new cases. For South and Southeast Asia, where the disease burden was increasing, expanding healthcare coverage, strengthening health education, and improving screening rates will be essential to curb the trend. Additionally, as smoking and high alcohol consumption remain among the leading risk factors (26), more stringent public health policies and focused interventions were necessary, particularly for high-burden and high-risk populations. Looking ahead, fostering transnational cooperation and providing technical supported to low SDI countries can significantly enhance their capacity for disease prevention and control. By leveraging shared knowledge, resources, and expertise, these collaborative efforts can contribute to further reducing the EC burden across Asia, driving progress toward a healthier future for the region as a whole.

## Conclusion

The overall decline in the disease burden of EC in Asia underscores the significant impact of medical advancements and public health interventions. However, persistent regional and sex disparities remain critical challenges that demand urgent attention. Addressing these inequalities will require multi-level and cross-sector collaboration, with a particular focus on under-researched regions such as South and Southeast Asia, where epidemiological trends and healthcare access patterns remain insufficiently explored.

Future efforts should prioritize strengthening healthcare resources in under-resourced and high-risk areas while refining prevention and control strategies to enhance their effectiveness. Additionally, targeted health policies tailored to the specific needs of high-risk populations and regions will be essential. Further research is needed to better understand the unique risk factors, healthcare barriers, and intervention effectiveness in South and Southeast Asia, where socioeconomic and cultural factors may influence disease progression differently than in other regions.

By integrating these strategies—along with region-specific research—it will be possible not only to further reduce the overall disease burden but also to bridge gaps in healthcare equity, fostering a more balanced and inclusive public health approach across Asia.

## Data Availability

Publicly available datasets were analyzed in this study. This data can be found here: Global Burden of Disease database, https://vizhub.healthdata.org/gbd-results/.

## References

[1] Wang W Ye L Li H Mao W Xu X. Targeting esophageal carcinoma: molecular mechanisms and clinical studies. MedComm (2020). (2024) 5:e782. doi: 10.1002/mco2.782 39415846 PMC11480525

[2] Collaborators GBDOC. The global, regional, and national burden of oesophageal cancer and its attributable risk factors in 195 countries and territories, 1990-2017: A systematic analysis for the global burden of disease study 2017. Lancet Gastroenterol Hepatol. (2020) 5:582–97. doi: 10.1016/S2468-1253(20)30007-8 PMC7232026 32246941

[3] Bandidwattanawong C. Multi-disciplinary management of esophageal carcinoma: current practices and future directions. Crit Rev Oncol Hematol. (2024) 197:104315. doi: 10.1016/j.critrevonc.2024.104315 38462149

[4] Yao L Wang X Wang Z Wang X Guo X. Expression and functional analyses of terf2 in esophageal carcinoma. Heliyon. (2024) 10:e38040. doi: 10.1016/j.heliyon.2024.e38040 39328506 PMC11425175

[5] Huang K Zhang B Feng Y Ma H. Magnolol promotes the autophagy of esophageal carcinoma cells by upregulating hace1 gene expression. Acta Biochim Biophys Sin (Shanghai). (2024) 56:1044–54. doi: 10.3724/abbs.2024044 PMC11322865 38660717

[6] Lopez-Gomez M Morales M Fuerte R Munoz M Delgado-Lopez PD Gomez-Cerezo JF. Prevalence of helicobacter pylori infection among patients with esophageal carcinoma. World J Gastroenterol. (2024) 30:3479–87. doi: 10.3748/wjg.v30.i29.3479 PMC11326089 39156503

[7] Murray CJL Collaborators GBD. Findings from the global burden of disease study 2021. Lancet. (2024) 403:2259–62. doi: 10.1016/S0140-6736(24)00769-4 38762327

[8] Jiang D Wu Y Liu L Shen Y Li T Lu Y. Burden of gastrointestinal tumors in asian countries, 1990-2021: an analysis for the global burden of disease study 2021. Clin Epidemiol. (2024) 16:587–601. doi: 10.2147/CLEP.S472553 39252850 PMC11381218

[9] Qi L Sun M Liu W Zhang X Yu Y Tian Z. Global esophageal cancer epidemiology in 2022 and predictions for 2050: A comprehensive analysis and projections based on globocan data. Chin Med J (Engl). (2024) 137:3108–16. doi: 10.1097/CM9.0000000000003420 PMC11706580 39668405

[10] Mazidimoradi A Amiri S Khani Y Allahqoli L Salehiniya H. Burden of esophageal cancer between 2010 and 2019 in asian countries by geographical region and sociodemographic index: A comparison with global data. Thorac Cancer. (2023) 14:2361–407. doi: 10.1111/1759-7714.15026 PMC10447175 37455657

[11] Ahuja P Yadav R Goyal S Yadav C Ranga S Kadian L. Targeting epigenetic deregulations for the management of esophageal carcinoma: recent advances and emerging approaches. Cell Biol Toxicol. (2023) 39:2437–65. doi: 10.1007/s10565-023-09818-5 37338772

[12] Morgan E Soerjomataram I Rumgay H Coleman HG Thrift AP Vignat J. The global landscape of esophageal squamous cell carcinoma and esophageal adenocarcinoma incidence and mortality in 2020 and projections to 2040: new estimates from globocan 2020. Gastroenterology. (2022) 163:649–58.e2. doi: 10.1053/j.gastro.2022.05.054 35671803

[13] Li P Jing J Liu W Wang J Qi X Zhang G. Spatiotemporal patterns of esophageal cancer burden attributable to behavioral, metabolic, and dietary risk factors from 1990 to 2019: longitudinal observational study. JMIR Public Health Surveill. (2023) 9:e46051. doi: 10.2196/46051 37801354 PMC10589835

[14] Xi Y Shen Y Chen L Tan L Shen W Niu X. Exosome-mediated metabolic reprogramming: implications in esophageal carcinoma progression and tumor microenvironment remodeling. Cytokine Growth Factor Rev. (2023) 73:78–92. doi: 10.1016/j.cytogfr.2023.08.010 37696716

[15] Chen C Chen S Cao H Wang J Wen T Hu X. Prognostic significance of autophagy-related genes within esophageal carcinoma. BMC Cancer. (2020) 20:797. doi: 10.1186/s12885-020-07303-4 32831056 PMC7446118

[16] Chen Z Zhang X Zhai J Fan J Cai Y Ye T. Global burden of esophageal cancer attributable to high bmi in 204 countries and territories: 1990-2019. Thorac Cancer. (2024) 15:681–92. doi: 10.1111/1759-7714.15239 PMC10961222 38316627

[17] Jin W Huang K Ding Z Zhang M Li C Yuan Z. Global, regional, and national burden of esophageal cancer: A systematic analysis of the global burden of disease study 2021. biomark Res. (2025) 13:3. doi: 10.1186/s40364-024-00718-2 39762900 PMC11702276

[18] Hong MZ Li JM Chen ZJ Lin XY Pan JS Gong LL. Global burden of major gastrointestinal cancers and its association with socioeconomics, 1990-2019. Front Oncol. (2022) 12:942035. doi: 10.3389/fonc.2022.942035 36387124 PMC9664003

[19] Lu L Mullins CS Schafmayer C Zeissig S Linnebacher M. A global assessment of recent trends in gastrointestinal cancer and lifestyle-associated risk factors. Cancer Commun (Lond). (2021) 41:1137–51. doi: 10.1002/cac2.12220 PMC8626600 34563100

[20] Fan J Liu Z Mao X Tong X Zhang T Suo C. Global trends in the incidence and mortality of esophageal cancer from 1990 to 2017. Cancer Med. (2020) 9:6875–87. doi: 10.1002/cam4.3338 PMC7520289 32750217

[21] Mazidimoradi A Banakar N Khani Y Allahqoli L Salehiniya H. Current status and temporal trend in incidence, death, and burden of esophageal cancer from 1990-2019. Thorac Cancer. (2023) 14:2408–58. doi: 10.1111/1759-7714.15028 PMC10447176 37443420

[22] Teng Y Xia C Cao M Yang F Yan X He S. Esophageal cancer global burden profiles, trends, and contributors. Cancer Biol Med. (2024) 21:656–66. doi: 10.20892/j.issn.2095-3941.2024.0145 PMC11359494 39066471

[23] Li W Wang W. Contribution of high body mass index to the global burden of esophageal cancer: A population-based study from 1990 to 2019. Dig Dis Sci. (2024) 69:1125–34. doi: 10.1007/s10620-024-08290-3 38433126

[24] Africa GBDN, the Middle East Neurology C. The burden of neurological conditions in North Africa and the middle east, 1990-2019: A systematic analysis of the global burden of disease study 2019. Lancet Glob Health. (2024) 12:e960–e82. doi: 10.1016/S2214-109X(24)00093-7 PMC11099299 38604203

[25] Andrici J Eslick GD. Hot food and beverage consumption and the risk of esophageal cancer: A meta-analysis. Am J Prev Med. (2015) 49:952–60. doi: 10.1016/j.amepre.2015.07.023 26590941

[26] Wu S Jiang W Li J Wu Z Xu C Xie N. Global burden of esophageal cancer attributable to smoking: A systematic analysis for the global burden of disease study 2019. Front Oncol. (2023) 13:1223164. doi: 10.3389/fonc.2023.1223164 37621692 PMC10446760

