# Supplementary material for: Current trends and future projection for addressing the burden of esophageal carcinoma in Asia: a comprehensive analysis (1990–2040)
Source: Front Oncol. 2025 Jun 23;15:1587846. doi: 10.3389/fonc.2025.1587846 (PMC12230575; doi:10.3389/fonc.2025.1587846)
Supplement: Supplementary file 1 [file Table1.docx]

Supplementary Table 1. Countries included in the study.

| Country | Country | Country |
| --- | --- | --- |
| Afghanistan | Kuwait | Thailand |
| Armenia | Kyrgyzstan | Timor-Leste |
| Azerbaijan | Lao People’s Democratic Republic | Turkmenistan |
| Bahrain | Lebanon | Turkey |
| Bangladesh | Malaysia | United Arab Emirates |
| Bhutan | Maldives | Uzbekistan |
| Brunei Darussalam | Mongolia | Viet Nam |
| Cambodia | Myanmar | Yemen |
| China | Nepal |  |
| Cyprus | Oman |  |
| Democratic People’s Republic of Korea | Pakistan |  |
| Georgia | Palestine |  |
| India | Philippines |  |
| Indonesia | Qatar |  |
| Iran (Islamic Republic of) | Republic of Korea |  |
| Iraq | Saudi Arabia |  |
| Israel | Singapore |  |
| Japan | Sri Lanka |  |
| Jordan | Syrian Arab Republic |  |
| Kazakhstan | Tajikistan |  |

Supplementary Table 2. The global burden of EC in different regions or sex.

| Location | Sex | 1990 ASR (95% CI) | 2021 ASR (95% CI) | EAPC_95%CI |
| --- | --- | --- | --- | --- |
| Prevalence | | | | |
| Central Asia | Male | 24.09 (22.92-25.39) | 7.81 (7.00-8.76) | -3.85 |
|  | Female | 13.04 (12.29-13.69) | 4.67 (4.08-5.37) | -3.36 |
| East Asia | Male | 48.97 (40.31-58.59) | 38.39 (29.75-48.32) | -0.81 |
|  | Female | 19.48 (10.05-24.57) | 11.88 (7.01-15.64) | -1.7 |
| South Asia | Male | 5.82 (5.08-6.67) | 5.58 (4.87-6.35) | -0.13 |
|  | Female | 5.12 (4.19-7.40) | 4.03 (3.35-5.71) | -0.8 |
| Southeast Asia | Male | 4.88 (4.04-6.07) | 5.56 (4.75-6.42) | 0.44 |
|  | Female | 2.85 (2.04-3.43) | 2.10 (1.67-2.55) | -1.03 |
| Asia | Male | 26.52 (22.81-30.73) | 22.26 (18.37-26.59) | -0.59 |
|  | Female | 11.39 (7.09-13.55) | 7.42 (5.28-9.15) | -1.45 |
| Incidence | | | | |
| Central Asia | Male | 18.03 (17.04-19.11) | 5.98 (5.38-6.66) | -3.64 |
|  | Female | 9.38 (8.86-9.84) | 3.31 (2.92-3.75) | -3.41 |
| East Asia | Male | 35.34 (29.39-41.95) | 24.49 (19.11-30.67) | -1.24 |
|  | Female | 14.03 (7.61-17.44) | 6.26 (3.72-8.24) | -2.74 |
| South Asia | Male | 4.19 (3.62-4.81) | 3.97 (3.47-4.50) | -0.18 |
|  | Female | 3.63 (2.94-5.25) | 2.78 (2.31-3.99) | -0.9 |
| Southeast Asia | Male | 3.46 (2.88-3.95) | 3.62 (3.14-4.13) | 0.15 |
|  | Female | 2.00 (1.46-2.41) | 1.37 (1.11-1.68) | -1.27 |
| Asia | Male | 18.08 (15.57-20.91) | 13.50 (11.13-16.12) | -0.99 |
|  | Female | 7.92 (5.02-9.43) | 4.05 (2.89-4.96) | -2.24 |
| Deaths | | | | |
| Central Asia | Male | 19.40 (18.29-20.57) | 6.47 (5.84-7.22) | -3.57 |
|  | Female | 10.09 (9.54-10.61) | 3.54 (3.14-4.00) | -3.38 |
| East Asia | Male | 37.38 (31.28-44.25) | 23.50 (18.38-29.23) | -1.58 |
|  | Female | 14.81 (8.16-18.31) | 5.66 (3.36-7.37) | -3.23 |
| South Asia | Male | 4.45 (3.83-5.11) | 4.21 (3.69-4.76) | -0.18 |
|  | Female | 3.85 (3.13-5.58) | 2.91 (2.41-4.21) | -0.94 |
| Southeast Asia | Male | 3.64 (3.04-4.49) | 3.65 (3.17-4.16) | 0.01 |
|  | Female | 2.12 (1.56-2.55) | 1.40 (1.13-1.73) | -1.4 |
| Asia | Male | 18.66 (16.10-21.59) | 12.80 (10.57-15.26) | -1.27 |
|  | Female | 8.25 (5.29-9.79) | 3.76 (2.71-4.58) | -2.64 |
| DALYs | | | | |
| Central Asia | Male | 480.28 (457.47-505.77) | 152.78 (136.65-170.86) | -3.89 |
|  | Female | 250.35 (236.06-262.99) | 87.39 (75.90-101.01) | -3.42 |
| East Asia | Male | 944.04 (779.25-1126.87) | 530.08 (408.64-668.77) | -1.94 |
|  | Female | 338.84 (176.89-424.82) | 112.38 (69.88-146.80) | -3.72 |
| South Asia | Male | 118.12 (102.96-135.84) | 107.57 (93.72-122.39) | -0.31 |
|  | Female | 102.04 (83.29-148.39) | 75.14 (62.33-106.87) | -1.05 |
| Southeast Asia | Male | 95.50 (79.39-118.59) | 93.74 (80.64-107.49) | -0.06 |
|  | Female | 54.04 (38.88-64.99) | 32.98 (26.54-41.11) | -1.69 |
| Asia | Male | 482.13 (410.86-562.90) | 292.07 (238.76-351.22) | -1.73 |
|  | Female | 194.88 (120.88-232.47) | 81.94 (63.17-98.48) | -2.92 |
